# Characterization of Patients with COVID-19 Admitted to a Community Hospital of East Harlem in New York City

**DOI:** 10.7759/cureus.9836

**Published:** 2020-08-18

**Authors:** Ahmed Shady, Ajay P Singh, Ejiro Gbaje, Marlon Oliva, Samantha Golden-Espinal, Dylan Macciola, Dyanna Soto, William E. Eddy, Anusha Adkoli, Nora V Bergasa

**Affiliations:** 1 Department of Medicine, NYC Health + Hospitals / Metropolitan, New York, USA; 2 Department of Medicine, New York Medical College, Valhalla, USA; 3 Department of Medicine, Physician Affiliate Group of New York, New York, USA

**Keywords:** coronavirus disease 2019 (covid-19), covid, covid-19, corona, pandemic, new york city, covid nyc

## Abstract

Background

New York City was the epicenter for the coronavirus disease 2019 (COVID-19) in the United States. Accordingly, the aim of this study was to characterize the population of patients admitted with this condition to a community hospital in East Harlem located in the northeast part of the city.

Methods

A retrospective review of medical records of patients at least 18 years of age, admitted to the hospital with COVID-19 disease from March 14 to April 30 of 2020.

Results

Three hundred and seventy-one patients were identified. The majority was comprised of men. Obesity, hypertension, and hyperlipidemia were the most prevalent comorbidities. Most patients were treated with a combination of hydroxychloroquine, azithromycin, zinc, and vitamin C. Twenty-three percent of the patients died from the disease during the study period.

Conclusion

Morbidity and mortality were substantial in patients with COVID-19 admitted to a community hospital in East Harlem.

## Introduction

The first documented coronavirus disease 2019 (COVID-19) case was in Wuhan, China, in the beginning of December 2019 [1]; this disease, caused by the severe acute respiratory syndrome coronavirus 2 (SARS-CoV-2), rapidly spread and was designated as a pandemic by the World Health Organization on March 11 of 2020 [2]. The first documented case in New York State was in early March of 2020 [3]; in a few weeks, New York City became the epicenter of this disease. Accordingly, the aim of this study was to characterize the patient population admitted with COVID-19 to a community hospital in East Harlem.

## Materials and methods

The study was a retrospective and a prospective review of the electronic medical records of patients aged at least 18 years admitted with findings suggestive of and with subsequently confirmed COVID-19 from March 14, 2020, when the first patient was admitted, to April 30, 2020. The study was approved by the Institutional Review Board.

Data were analyzed by the use of Sata version 16 (SataCorp, College Station, Texas, USA). Univariate statistics of non-missing observations were calculated for all variables. Means and 95% confidence intervals (CI) were calculated for continuous variables, and if they were found to be non-normal, medians, and interquartile ranges were computed. Continuous vital signs and laboratory measurements as well as body mass index (BMI) were categorized by the use of defined normal limits. Frequencies and percentages were determined for categorical variables.

## Results

Three hundred and seventy-one patients were admitted with COVID-19 disease; 67% were men, with the largest age group being from 55 to 64 years which comprised 23% of the total patient population (Table 1). Fifty-two percent of the subjects were characterized as non-Hispanic, although the information on race and ethnicity was limited. Twelve patients were pregnant. 

**Table 1:** Characteristics of patients with COVID-19 (N = 371)

Demographics		
	N	%
Total	371	100
Age in years, median (IQR)	57	(42, 68)
Age in years		
18-34	40	10.78
35-44	61	16.44
45-54	67	18.06
55-64	87	23.45
65-74	66	17.79
>75	50	13.48
Gender		
Male	249	67.12
Female	122	32.88
Comorbidities		
	N	%
Asthma (1 missing)	42	11.35
Chronic Obstructive Pulmonary Disease (1 missing)	21	5.68
Hypertension (1 missing)	182	49.19
Hyperlipidemia (3 missing)	101	27.45
Congestive Heart Failure (3 missing)	28	7.61
Diabetes Mellitus (1 missing)	156	42.16
HIV (2 missing)	7	1.90
Rheumatological Disease (1 missing)	7	1.89
End Stage Renal Disease (1 missing)	18	4.86
Chronic Kidney Disease (1 missing)	45	12.16
Cirrhosis (1 missing)	8	2.16
Immunosuppression (1 missing)	111	30.00
Pregnancy		
	N	%
Pregnant Women	12	3.50
BMI (37 missing)		
	N	%
Underweight (<18.5)	7	2.10
Normal (18.5-<25)	73	21.86
Overweight (25-<30)	109	32.63
Obesity Level 1 (30-<35)	82	24.55
Obesity Level 2 (35-<40)	32	9.58
Obesity Level 3 (> 40)	31	9.28
Vital Signs		
	N	%
Patient’s temperature at presentation, degrees Fahrenheit, median (IQR) (9 missing)	99.40	(98.40, 101.10)
Patient’s maximum temperature during hospital stay, degrees Fahrenheit, median (IQR) (1 missing)	100.80	(99.40, 102.20)
Patient’s minimum temperature during hospital stay, degrees Fahrenheit, median (IQR) (1 missing)	97.60	(97.20, 98.00)
Tachycardia	185	49.87
Systolic Blood Pressure on Presentation (3 missing)		
<100 mmHg	28	7.61
>100 mmHg	340	92.39
Percent Oxygen Saturation on Presentation		
<88%	51	13.75
88-92%	75	20.22
>92%	245	66.04
Respiratory Rate (10 missing)		
< 20 rpm (Normal)	236	65.37
>20 rpm (Abnormal)	125	34.63
Symptoms		
	N	%
Cough (8 missing)	271	74.66
Shortness of Breath (8 missing)	265	73.00
Fever (7 missing)	247	67.86
Body Aches (13 missing)	118	32.96
Diarrhea (14 missing)	77	21.57
Nausea (15 missing)	63	17.70
Vomiting (13 missing)	48	13.41
Abdominal Pain (15 missing)	40	11.24
Sick Contacts (88 missing)	45	15.90
Recent Travel (107 missing)	7	2.65
Laboratory Results		
	N	%
pH (89 missing)		
Low (<7.3)	34	12.06
Normal (7.3-7.4)	133	47.16
High (>7.4)	115	40.78
pCO_2_ (89 missing)		
Low (<35 mmHg)	62	21.99
Normal (35-48 mmHg)	178	63.12
High (>48 mmHg)	42	14.89
White Blood Cell count		
Low (<4.8 x 10^3^ cells/mcL)	64	17.25
Normal (4.8 x 10^3^ - 10.8 x 10^3^ cells/mcL)	206	55.53
High (>10.8 x 10^3^ cells/mcL)	101	27.22
Lymphopenia (1 missing)	302	81.62
Platelet count		
Low (<150 x 10^3^ count/mcL)	77	20.75
Normal (150 x 10^3^ – 450 x 10^3^ count/mcL)	275	74.12
High (>450 x 10^3^ count/mcL)	19	5.12
Hemoglobin level (Male) (122 missing)		
Male Low (<14 g/dL)	130	52.21
Male Normal (14-18 g/dL)	113	45.38
Male High (> 18g/dL)	6	2.41
Hemoglobin level (Female) (249 missing)		
Female Low (<12 g/dL)	55	45.08
Female Normal (12-16 g/dL)	64	52.46
Female High (>16 g/dL)	3	2.46
Sodium level (1 missing)		
Low (<136 mEq/L)	213	57.57
Normal (136-145 mEq/L)	149	40.27
High (>145 mEq/L)	8	2.16
Blood Urea Nitrogen level (1 missing)		
Normal (<20 mg/dL)	134	36.22
Abnormal (>20 mg/dL)	236	63.78
Serum Creatinine level (1 missing)		
Normal (<0.9 mg/dL)	152	41.08
Abnormal (>0.9 mg/dL)	218	58.92
Normal Baseline liver profile (185 missing)	118	63.44
Total Bilirubin (11 missing)		
Normal	324	90.00
Abnormal	36	10.00
Alkaline Phosphatase level (10 missing)		
Normal (<116 U/L)	284	78.67
Abnormal (>116 U/L)	77	21.33
Aspartate Aminotransferase level (10 missing)		
Normal (<30 U/L)	82	22.71
Abnormal (>30 U/L)	279	77.29
Alanine Aminotransferase (12 missing)		
Normal (<30 U/L)	162	45.13
Abnormal >30 U/L)	197	54.87
Creatine phosphokinase level (279 missing)		
Normal (<200 U/L)	50	54.35
Abnormal (>200 U/L)	42	45.65
Blood Lactate level (154 missing)		
Normal (<2.0 mmol/L)	152	70.05
Abnormal (>2.0 mmol/L)	65	29.95
Chronic Hepatitis B Status (259 missing)		
Positive	2	1.79
Negative	110	98.21
Chronic Hepatitis C Status (260 missing)		
Positive	8	7.21
Negative	103	92.79
Ferritin (105 missing)		
Normal (<400 ng/mL)	63	23.68
Abnormal (>400 ng/mL)	203	76.32
Pro B Type Natriuretic Peptide level (197 missing)		
Normal (<125.0 pg/mL)	61	35.06
Abnormal (>125.0 pg/mL)	113	64.94
Procalcitonin level (95 missing)		
Normal (<0.08 ng/mL)	40	14.49
Abnormal (>0.08 ng/mL)	236	85.51
D-dimer level (90 missing)		
Normal (<230 ng/dL)	59	21.00
Abnormal (>230 ng/dL)	222	79.00
HbA1c (Percentage of Glycosylated Hb) (294 missing)*		
Non-Diabetic + Controlled Diabetic (< 8 %)	48	62.34
Uncontrolled Diabetic (>8% )	29	37.66
Note: Some frequencies do not add up to the total 371 due to missing observations *HbA1C at least within the prior three months of admission		

The most common symptoms at presentation were cough in 75% of the patients, shortness of breath in 73%, body aches in 33%, and fever in 68%; others included diarrhea, in 22%, and nausea and abdominal pain.

The majority of subjects, 92%, presented with a systolic blood pressure above 100 mmHg and normal oxygen saturation, with 50% exhibiting tachycardia.

Notable comorbidities were asthma or chronic obstructive pulmonary disease (COPD) in 16% of the patients, hypertension in 49%, hyperlipidemia in 27%, and diabetes in 42%.

A body mass index (BMI) of more than 25 kg/m2 was documented in 76% of the subjects. A BMI between 25 and 30 kg/m^2^, consistent with the definition of overweight, was documented in 33% of the patients; obesity class 1 was documented in 25%, class 2 in 10%, and class 3 in 9%.

At presentation, a normal pH was exhibited by 47% of the patients, with alkalemia noted in 41%. Lymphopenia was present in 82% of the subjects, 58% had hyponatremia, and 58% had a serum creatinine >0.9 mg/dL. Increased activity of serum aspartate aminotransferase was present in 77%, with 46% exhibiting elevated serum creatine phosphokinase. Serum lactate was high in 30% of the subjects, procalcitonin in 86%, and D-dimer in 79%. Serum ferritin was greater than 400 mg/ml, more than twice the upper limit of normal, in 76% of the group.

Seventy-seven patients had hemoglobin A1c (HbA1c) percentage documented in their chart at least three months prior to admission; of those, 38% had uncontrolled diabetes with HbA1c > 8%. Acute kidney injury was documented in the charts of 31% of the group.

During hospitalization, 32% of the patients required admission to the intensive care unit (ICU), 25% were intubated, 20% required vasopressor support, and 30% had a diagnosis consistent with acute respiratory distress syndrome (ARDS).

The majority of patients, 94%, were treated with hydroxychloroquine, 93% with azithromycin, most with a combination of both; in addition, 88% received zinc, and 89% vitamin C. Steroids, lopinavir/ritonavir, tocilizumab, and therapeutic anticoagulation were administered to 21%, 7%, 14%, and 21%, respectively. Inpatient mortality was 23%.

## Discussion

Over the study period, 371 patients, the majority of whom were men, with a median age of 57, were hospitalized with COVID-19. More than two thirds of the patients had an elevated BMI. Hypertension and diabetes mellitus were the most common comorbidities. Laboratory findings were similar to what has been reported in other studies [1,4-6]. More than one-fourth of the patients developed acute kidney injury, ARDS and required ICU care, and one fourth required mechanical ventilation. The majority of the patients received hydroxychloroquine, azithromycin, zinc, and vitamin C, consistent with the treatment being proposed at the time. The mortality was 23%.

A salient finding of this study was that in 76% of the patients, the BMI was equal to or greater than 25 Kg/m2, with 43% of this group having a BMI equal to or greater than 30 Kg/m2, consistent with obesity; this figure exceeds the prevalence of obesity in New York City, which is reported in 35.1% of its population [7], and also, it is higher than what has been reported in other studies related to COVID-19 [4,8]. This finding is consistent with the concern that obesity may be a risk factor for adverse outcomes in patients with COVID-19 [9]; this observation may be secondary to the fact that obesity propitiates the development of an inflammatory state, as suggested by elevated serum levels of some pro-inflammatory proteins in some obese subjects [10]. In this context, the high serum lactate and procalcitonin concentrations in 30% and 86% of patients, respectively, are consistent with a high inflammatory milieu in association with this disease. 

The most common symptoms at presentation concerned the respiratory system, consistent with what has been documented in other studies [1,4,5]. However, diarrhea was reported by 21% of the patients, which is higher than what has been previously reported, 3%-5% [1,5,6], although similar to what was documented from other studies from New York City, 23% [8]. This finding may point towards some regional variation in symptoms of patients with COVID-19, which may be related to contracting different strains of the virus, which may have increased affinity for the ACE 2 receptor on the enterocytes [11]. In this regard, the SARS-COV2 virus uses its transmembrane glycoprotein to attach to the ACE 2 receptors via the transmembrane protease (TMPRSS). In addition to the lungs, the ACE 2 receptors are expressed by the esophagus, distal small intestine, and colon [12], all of which may be relevant in the gastrointestinal manifestations of COVID-19 [13]. Several hypotheses have been proposed to explain the pathogenesis of diarrhea in COVID-19, including increased permeability of the intestinal membrane, alteration of gut microbiome, and enzyme modifications leading to the development of colitis [11]. 

Hepatitis B (HBV) and C serologies were not available in 69% and 70% of the patients, respectively. In this context, we recommend that HBV serology be included as part of the initial laboratory investigations of patients with COVID-19, as treatment with tocilizumab, an immunosuppressant agent, is a drug being considered as a therapeutic option for this disease, and it may be associated with HBV reactivation [14]. Accordingly, treatment with oral antiviral medications against HBV may have to be considered prior to treatment with an immunosuppressant drug. The use of tocilizumab in patients with chronic hepatitis C does not appear to be associated with increased HCV replication or hepatocellular injury [15].

The majority of patients had high serum ferritin concentrations, consistent with secondary hemophagocytic lymphohistiocytosis, a syndrome characterized by a severe inflammatory response, usually to a viral infection, associated with massive hypercytokinemia and multiorgan failure, and which was the basis for the use of steroids in the treatment of some of the patients in this study [16].

Acute kidney injury was present in 31% of the patients, which is significantly higher than that reported in other studies [4,6]. A hypercoagulable state has been implicated in the pathogenesis of renal disease in patients with SARS-COV2 infection. Autopsy reports have documented the presence of proximal tubular necrosis and glomerular fibrin thrombi in patients with COVID-19 who developed acute kidney injury [17]. In our study, D-dimer plasma concentrations were elevated in 79% of the patients, 27% of whom received therapeutic anticoagulation, in congruence with the prevailing understanding that COVID-19 is a pro-thrombotic condition. 

Mechanical ventilation was required by 25% of the patients; this proportion was slightly higher than that reported in other studies [4,6,8]. In addition, over a short study period of 48 days, the mortality was high, 23%. However, this may not represent the true mortality rate as 45 patients were still hospitalized at the time data collection was completed for this study. 

Median length of stay in the hospital in patients who died or were discharged was seven days, which is less than that reported in studies from China [6], but more than that reported from other studies from New York City [4]. Similar length of stay for surviving and deceased patients may suggest that the disease can progress rapidly in some and also, that individuals who make it through a certain period of time, e.g. seven days, without deterioration have a good probability of surviving. In this context, survival may be influenced by comorbidities, and by genetic susceptibility. In this regard, a limitation of this study was the missing data from several categories including race and ethnicity, which may impact the generalizability of this study as well as the identification of areas for future investigation.

## Conclusions

In conclusion, COVID-19 in the patients from the community of East Harlem in New York City, comprised mostly of African or Hispanic heritage population, was associated with severe disease and high mortality. The presenting symptoms in the patients studied were similar to those reported from other parts of the world; however, diarrhea had an increased incidence in this study group. The features associated with the substantial mortality documented may include a high BMI; in this context, obesity and genetic susceptibility warrant investigation as factors that contribute to the natural history of COVID-19.
